# Assessment of hemodynamic and blood parameters that may reflect macroscopic quality of porcine kidneys during normothermic machine perfusion using whole blood

**DOI:** 10.1007/s00345-024-05139-2

**Published:** 2024-08-07

**Authors:** Carla Steinhauser, Abdulbaki Emre Yakac, Wenke Markgraf, Susanne Kromnik, Andreas Döcke, Philipp Talhofer, Christine Thiele, Hagen Malberg, Susanne Füssel, Christian Thomas, Juliane Putz

**Affiliations:** 1 Department of Urology, Technische Universität Dresden, Fetscherstraße 74, 01307 Dresden, Germany; 2 Institute of Biomedical Engineering, Technische Universität Dresden, Fetscherstraße 29, 01307 Dresden, Germany

**Keywords:** Kidney, Normothermic machine perfusion, Organ assessment, Pig, Whole blood, Reperfusion

## Abstract

**Purpose:**

Using ex vivo normothermic machine perfusion (NMP) with whole blood we assessed marginal porcine kidneys under reperfusion. The aim was to link measureable machine and clinical blood parameters with the currently used visual assessment. This could serve as a baseline for a standardized evaluation score to identify potentially transplantable kidneys in the future.

**Methods:**

Kidneys and autologous whole blood were procured from slaughterhouse pigs (*n* = 33) and were perfused for 4 h using NMP. The hemodynamic parameters arterial pressure (AP), renal blood flow (RBF) and intrarenal resistance (IRR) were measured. Activity of aspartate transaminase (AST), gamma-glutamyltransferase (GGT), alkaline phosphatase (ALP), lactate dehydrogenase (LDH) and lactate were assessed in blood at 0/1/2/4 h. Kidneys were grouped into “potentially transplantable” (PT) or “not transplantable” (NT) based on their overall macroscopic appearance after NMP by an experienced physician.

**Results:**

PT-kidneys (*n* = 20) had a significantly lower IRR and higher RBF than NT-kidneys (*n* = 13). GGT, ALP and LDH did not differ significantly, but at 4 h, AST was significantly higher in PT-kidneys compared to NT-kidneys. Lactate levels kept increasing during NMP in NT-kidneys and were significantly higher at 1/2/4 h than in PT-kidneys.

**Conclusion:**

The immediately assessed macroscopic aspects of examined kidneys correlated with hemodynamic parameters, increased lactate and lower AST in this study. In the future, NMP with whole blood could be a useful tool to extend the donor pool by allowing the assessment of otherwise unknown characteristics of marginal kidneys before transplantation.

**Supplementary Information:**

The online version contains supplementary material available at 10.1007/s00345-024-05139-2.

## Introduction

Despite the need for transplantable kidneys, supply constantly falls short [1]. An ageing population results in an increased median age and comorbidities of deceased donors [1]. Some of these extended criteria donor (ECD) kidneys are discarded because the provided donor data does not allow an adequate assessment of the potential functionality of these organs, even though they might still be transplantable [2].

Hypothermic machine perfusion (HMP) has been well studied so far and demonstrated in systematic reviews a lower risk of delayed graft function (DGF) and primary non-function compared to the static cold storage (SCS) protocol [3, 4]. In a laboratory setting using pigs, normothermic machine perfusion (NMP) was shown to be favorable for graft function [5]. Machine perfusion allows various approaches with variation of temperature, perfusates and supplementation of oxygen [6]. NMP with oxygenated whole blood allows observation of the kidney under physiological conditions simulating reperfusion. However, most publications about NMP describe the usage of either packed or washed red blood cells [7–9].

There are approaches for identifying functional kidneys during NMP. Renal function was successfully evaluated using inulin clearance in a porcine model [10]. The same group found that the inulin clearance-based classification could accurately be reproduced using hyperspectral imaging and the convolutional neural network KidneyResNet [11]. These approaches, although of high quality and informative value, are difficult to implement in a clinical setting. In a recent promising study the group was also able to correlate, among others, perfusion hemodynamic parameters of laboratory pig kidneys with their inulin clearance, allowing for an assessment during NMP based on supervised machine learning methods [12]. Hosgood et al. developed an assessment score including macroscopic appearance, renal blood flow (RBF) and urine output based on discarded human kidneys [13]. They found that declined kidneys assessed by this score could be successfully transplanted [14].

Here, we describe the possibility of stable NMP with whole blood over 4 h in a preclinical animal model. The aim was to classify the organs macroscopically into “potentially transplantable” (PT) and “not transplantable” (NT), similarly to the current clinical practice, and to compare the classification with the recorded hemodynamic and blood parameters.

## Materials and methods

### Setting

Using the challenging approach of whole blood, we tried to closely mimic the environment of the kidneys after transplantation in the ischemia-reperfusion setting. This allows an early assessment of the kidney performance shortly after reperfusion and a pre-conditioning of the organ. In this study, slaughterhouse pigs were used as model to generate “marginal organs” due to longer and varying WIT. In this pre-clinical setting, whole blood was used as it is both easy to procure during the slaughterhouse process without needing additional compatible donor blood. Compared to washed or packed erythrocytes, whole blood still contains immune cells, allowing to observe a moderate immune response without the extra boost from allogenic blood. This could be different in a clinical setting later on. In this setting it was the best way to simulate a reperfusion in the closest model to real life. Of course blood from a human organ donor in the needed volume might not be possible. As NMP is not yet a standardized method, many perfusion durations can be found in the literature. One study found 4 h of perfusion optimal, as longer perfusion caused an increase of damage markers [15]. This corresponds to our further criteria of staying below 6 h of perfusion to be later adoptable in a clinical setting as well as to prevent too much blood dilution caused by urine repletion.

Therefore, thirty-three porcine kidneys were recovered during the slaughterhouse process (Dresden, Germany). Pigs were cross-breds of German Landrace, German Large White and Piétrain, weighing 105–150 kg. About 1 l of autologous blood was collected in a receptacle containing 5000 I.E. heparin before being transferred into two 500 ml-bottles with 70 ml 0.2 M citrate buffer pH 5.0, 5000 I.E. heparin and 3 ml 5% glucose solution. Kidneys were taken *en bloc* once the slaughterhouse process allowed and separated back-table. Initial flushing was done using 40 ml isotonic NaCl solution supplemented with 400 I.E. heparin. Kidneys were subsequently flushed up to 15 min with histidine-tryptophane-ketoglutarat (HTK) perfusion solution containing heparin (10,000 i.E./l) until effluent appeared clear. Blood bottles and flushed kidneys, packed bag-in-bag in HTK solution, were stored in iced water as SCS.

### Normothermic machine perfusion with whole blood

The kidneys were perfused in a self-constructed prototype of a NMP-device with whole blood (“nephroProtect”, project no. VP2904501KJ1, Institute of Biomedical Engineering, TU Dresden, Germany) [10]. The circuit allowed the perfusion of the kidneys with controlled temperature and pressure. Circulating blood was oxygenated (Oxygenator: Capiox FX05, Terumo, Eschborn, Germany) and supplied with glucose. Excreted urine volume was replenished with Ringer’s solution.

Detailed instructions for perfusion circuit set-up and handling were previously described by Markgraf et al. [10]. Briefly, the circuit of the perfusion device was primed using Ringer’s solution before the heparinized whole blood was added. The perfusate was conditioned to a target temperature of 37 °C, a glucose level of 5 mmol/l and a pH between 7.35 and 7.45. Afterwards, the kidneys were removed from SCS and joined to the circuit using connectors designed by the Institute of Biomedical Engineering (Dresden, Germany) and the perfusion was started. The RBF could be set manually to a certain value or automatically regulated within the physiological range. At the start of perfusion the RBF was set to 200 ml/min before allowing automated control. During automated control the perfusion circuit would attempt to adjust the flow rate to achieve an arterial pressure (AP) of 80 mmHg. The RBF (in ml/min), AP (in mmHg) and kidney temperature, assessed indirectly by venous blood temperature (in °C), were measured and recorded semi-continuously at 0.2 s-intervals. The intrarenal resistance (IRR) was calculated as the quotient of AP and RBF. RBF and IRR were normalized to pre-perfusion kidney weight. The recorded data points around the exact time point were averaged to avoid momentary fluctuations. For time point 0 h, all data points of the first 2 min were averaged; for 1 and 2 h, the 2 min before and after were averaged and for the 4 h time point, the 2 min before the end were averaged.

### Blood parameters

At 0 h, 1 h, 2 h and 4 h blood samples were collected. Activity of lactate dehydrogenase (LDH), alkaline phosphatase (ALP), gamma-glutamyltransferase (GGT), AST and lactate concentration were measured by standard procedures of clinical chemistry. Those markers were chosen from the routinely measurable markers due to their use for assessing kidney injury. LDH and AST were used as general cell injury markers, GGT and ALP as markers for tubule injury [16, 17].

### Immediate appraisal of kidney quality

After 4 h of NMP one present experienced urologist classified the kidneys non-blinded into two groups based on their overall macroscopic appearance. Kidneys with a homogeneous perfusion quality were considered PT, whereas kidneys with inhomogeneous perfusion or a livid discoloration were considered NT. The direct, visual appraisal was chosen to reflect daily clinical practice.

### Statistical analysis

Statistical analysis was carried out using GraphPad Prism 9.0 (GraphPad Software, San Diego, CA, USA). Where necessary, the mean value (mean) and standard deviation (SD) were calculated, To compare the two groups at each time point, Mann-Whitney U-test analysis was used.

## Results

### Kidney cohort

Based on their overall macroscopic appearance, 20 kidneys were considered PT and 13 kidneys were considered NT (S1). The warm ischemic times (WIT) did not differ between the PT- and NT-kidneys (respectively, 52.4 ± 12.9 vs. 54.4 ± 15.6 min; mean ± SD). The cold ischemic times (CIT) also did not vary (PT, *n* = 19 vs. NT, *n* = 13: 256.3 ± 162.7 min vs. 273.3 ± 171.3 min). WIT was defined as the time between death of the pig to SCS as well as preparation time before perfusion during which kidneys were no longer submerged under ice. CIT was defined as the total time the kidneys were stored under SCS. All kidneys had a continuous diuresis with no significant difference (PT, *n* = 20 vs. NT, *n* = 11: 7.1 ± 9.8 vs. 12.2 ± 11.3 ml/h/100 g; mean ± SD; *p* = 0.0868).

### Hemodynamic parameters during NMP

**Fig. 1:**
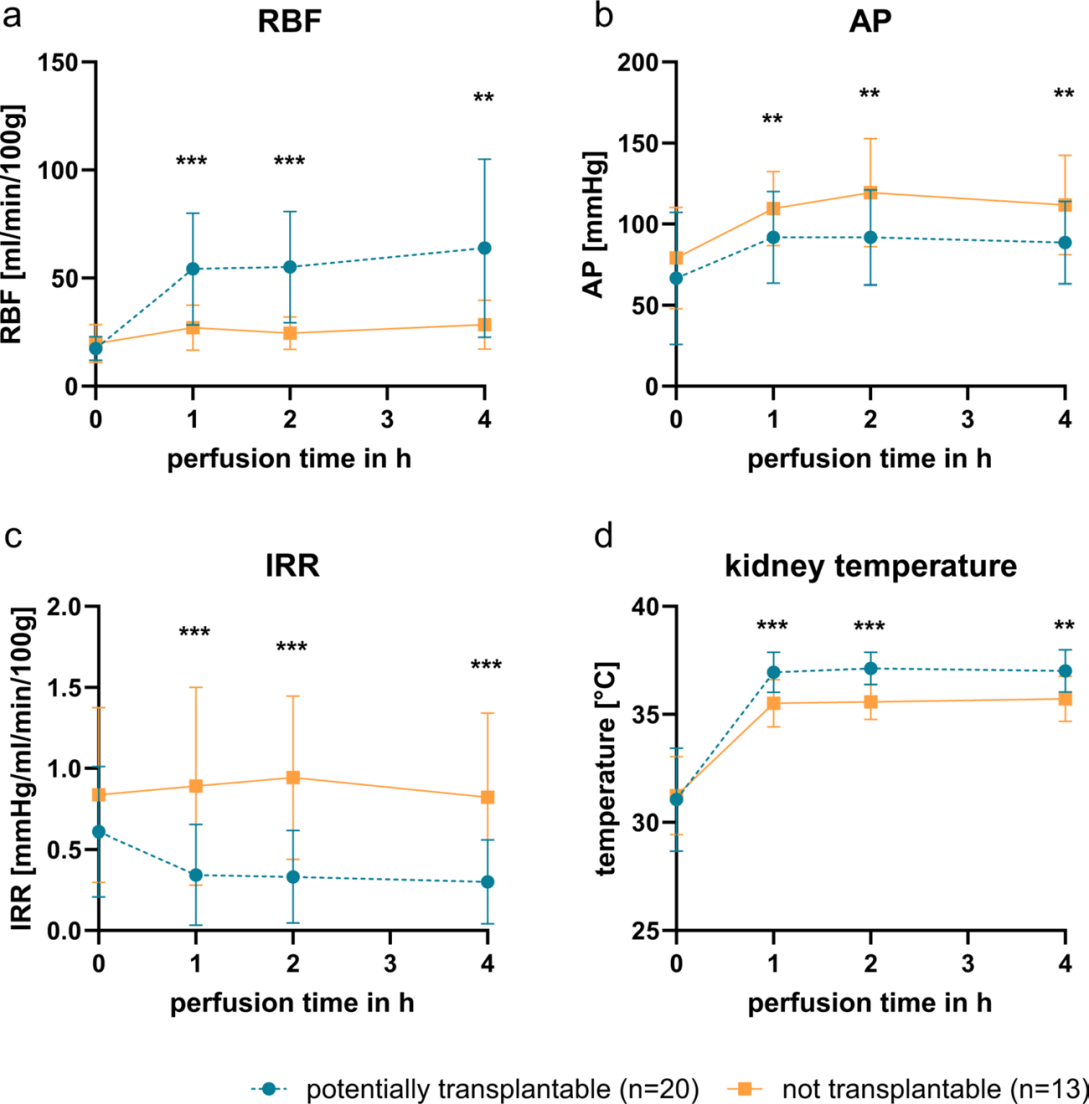
Course of the hemodynamic parameters during NMP. Throughout NMP the perfusion device semi-continuously recorded the renal blood flow (RBF) (**a**) and arterial pressure (AP) (**b**). From these two, the intrarenal resistance (IRR) was calculated (**c**). RBF and IRR were normalized to kidney weight. Additionally, the temperature of the kidneys was measured (**d**). To avoid distortion by temporary spikes, the mean of RBF, AP, IRR and temperature of the 2 min (for 0 h and 4 h) and 4 min (for 1 h and 2 h) time window around the time points was used. “Potentially transplantable” (blue lines, dashed lines, round points) were compared with the “Not transplantable” (orange, square points) kidneys. Depicted is the mean ± SD. Significance was calculated using Mann-Whitney-U-test. *p < 0.05 **p < 0.01 ***p < 0.001. Both groups started at similar levels in all parameters, but differed significantly after 1 h. The change in RBF, AP, IRR and temperature happened within the first hour. The levels in each group were then stable for the last 3 h of perfusion.

RBF was equal between both groups at 0 h NMP. It did not increase in NT-kidneys over time. However, in PT-kidneys the RBF improved within 1 h of NMP (54.23 ± 25.22 vs. 27.16 ± 10.03 ml/min/100 g; mean ± SD) and stayed at a higher level until the end. Both groups differed significantly at 1 h, 2 h (both *p* < 0.001) and 4 h (*p* = 0.0016) (Fig. 1a). The AP did not differ between the groups at 0 h. AP was significantly lower in PT-kidneys at 1 h, 2 h and 4 h compared to the NT-kidneys (e.g. at 4 h: 88.43 ± 24.79 vs. 111.67 ± 29.49 mmHg; *p* = 0.0025; mean ± SD) (Fig. 1b). The IRR was subsequently significantly different between the two groups as well (*p* < 0.001 at 1 h, 2 h, 4 h). In NT-kidneys, it stayed at a constant level of about 0.9 mmHg/ml/min/100 g during the entire NMP (Fig. 1c). On the contrary, the IRR decreased within the first hour of NMP in PT-kidneys (0.34 ± 0.3 vs. 0.89 ± 0.59 mmHg/ml/min/100 g; mean ± SD).

Kidney perfusate was cooled down by the cold kidneys at the start to an average temperature of 31.1 °C and warmed up within 1 h of NMP. However, only the perfusate of PT-kidneys reached the desired 37 °C (37.0 ± 0.95 °C at 4 h; mean ± SD). NT-kidneys’ perfusate only warmed up to a mean of 35.7 ± 1 °C even after 4 h of NMP. The difference was significant at all three time points (1 h and 2 h *p* < 0.001; 4 h *p* = 0.0012) (Fig. 1d).

### Blood parameters during NMP

**Fig. 2:**
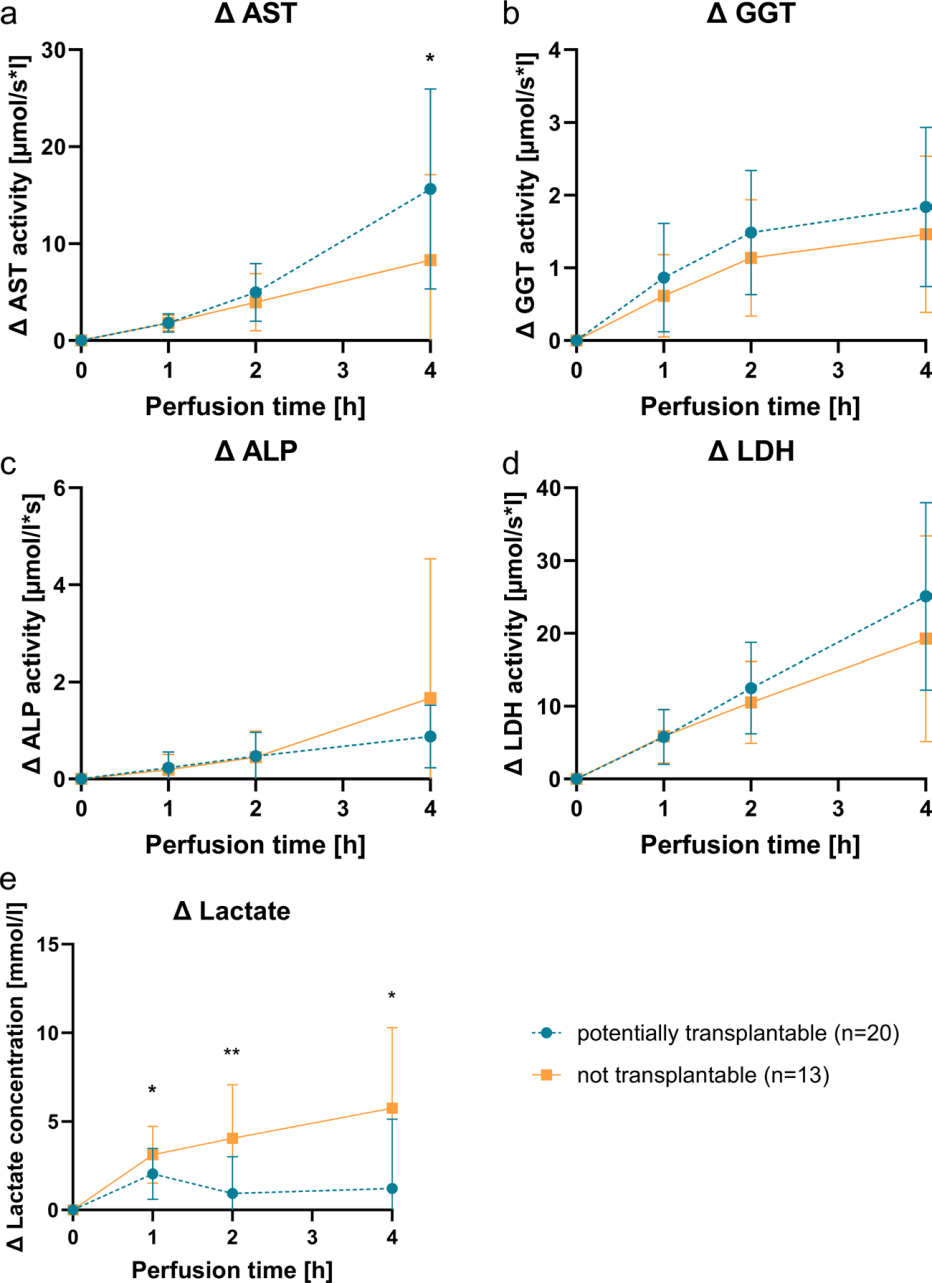
Course of the blood parameters during NMP. Throughout NMP the perfusion plasma samples were taken at 0, 1, 2 and 4 h. The activity of the parameters aspartate transaminase (AST) (**a**), Gamma-glutamyltransferase (GGT) (**b**), alkaline phosphatase (ALP) (**c**), lactate dehydrogenase (LDH) (**d**) and the concentration of lactate (**e**) were measured. The change from the initial value was calculated. “Potentially transplantable” (PT, blue dashed lines, round points) were compared with the “Not transplantable” (NT, orange lines, square points) kidneys. Depicted is the mean ± SD. Significance was calculated using Mann-Whitney-U-test. *p < 0.05 **p < 0.01 ***p < 0.001. All biomarker except lactate increased continuously over the 4 h of perfusion. The ΔAST levels at the 4 h mark were significantly higher in PT-kidneys than in NT kidneys. ΔGGT, ΔALP and ΔLDH did not differ between groups. In NT-kidneys Δlactate increased continuously during NMP. In PT-kidneys, however, Δlactate increased in the first hour before levels than declined from then on. The groups differed significantly at 1 h, 2 h and 4 h in Δlactate.

Activity levels of the blood parameters LDH, GGT, ALP, AST and concentration of lactate were measured and the difference to the initial value at 0 h was calculated (Δ concentration, Δ activity) to observe changes from the start of perfusion (Fig. 2).

LDH, ALP and GGT all increased over time in all kidneys. However, there was no significant difference between NT- and PT-kidneys. AST also increased over time and differed significantly at 4 h between the two groups. The AST increase was higher in PT-kidneys (*p* = 0.0243).

Lactate increased in both groups within 1 h of NMP. The increase was smaller in PT-kidneys (*p* = 0.0139). Lactate concentration kept increasing in NT-kidneys during the entire duration whereas the lactate concentration fell between the 1 h and 2 h time points in PT-kidneys. Both groups were significantly different at 1 h, 2 h and 4 h.

## Discussion

### Overall consideration

NMP with whole blood allows to observe kidneys before transplantation under almost physiological conditions. This may allow assessment of marginal kidneys. For this assessment parameters are needed that can be evaluated during or quickly after perfusion. For this purpose, 33 porcine kidneys were perfused and the hemodynamic parameters as well as a selection of clinical blood parameters were recorded. In this cohort, the macroscopic assessment of the kidneys reflected the perfusion parameters as we found elevated biomarker levels, higher IRR and AP as well as a lower RBF in NT-kidneys compared to PT-kidneys. While most other studies did not use whole blood, but erythrocyte-based perfusates, similar results to this study were reached [5, 7–9, 18–21]. This raises hopes that good post-transplant functionality of the grafts could be achieved using a physiological whole blood setting. We were able to reinforce the findings of Markgraf et al. that NMP with whole blood for 4 h is a feasible method to preserve kidneys [10].

### Hemodynamic parameters

Hemodynamic parameters can be monitored directly during perfusion, making them a direct and easy marker for assessing the kidneys. Specifically, the RBF, AP and IRR can give useful insight in the perfusion, most interestingly in its restriction. We found, that RBF was higher in PT-kidneys, allowing more blood to circulate in the kidneys. AP and IRR were higher in NT-kidneys, indicating an inhibited blood flow in NT-kidneys.

Studies done on smaller pigs found less tubular injury at 75 mmHg than at 55 mmHg in porcine kidneys during NMP with leucocyte-depleted blood [18] and better renal function at 95 mmHg compared to the lower pressures during NMP with whole blood [22]. This would agree with PT-kidneys leveling off at an AP of about 90 mmHg, with the exception of the pigs’ weight. However, the exact physiological range of reported blood pressure for pigs at varying weight differs greatly between studies [23–25].

The RBF of the PT-kidneys (54.2 ± 25.2 ml/min/100 g at 1 h) was comparable to that observed by Markgraf et al. in an equivalent NMP setting with laboratory pigs [10]. They identified a correlation between higher RBF and renal function [12]. Lignell et al. however set the AP to a constant 70 mmHg and observed an even higher RBF in porcine kidneys during NMP after SCS [9]. As a result, their observed IRR at 1 h NMP was higher than in our PT-kidneys, yet lower than in the NT-kidneys. While the absolute values differ, they also observed an initially high IRR that dropped within an hour, similar to the performance of the PT-kidneys [9]. This drop-off was also observed in similar NMP settings by other groups [5, 12] and in a study performing NMP using packed red blood cells on discarded human kidneys [7]. NT-kidneys were not able to lower their IRR in our study potentially predicting organ damage. Kaths et al. also described a correlation between a higher IRR at 1 h of NMP and a lower performance post-operation [8].

In NT-kidneys, the IRR stayed high even though the RBF was low, resulting in a lower volume of blood circulated through the kidneys. The insufficient blood flow was reflected by the kidney perfusate temperature, as kidneys were unable to warm up properly. This interrelation has also been discovered to be correlated to renal functionality in laboratory pigs [12]. This might have been caused by the formation of micro-thrombi that may have formed despite the addition of heparin to the blood and no detectable macro-thrombi in the main vessels. At the initial measurement, the cold kidneys performed similar, but once warmed up, PT-kidneys’ performance was superior. Moreover, once the kidneys had fully warmed up, the hemodynamic parameters were stable throughout the 4 h NMP.

The hemodynamic parameters reflected the perfusion quality. The context with the literature shows how the hemodynamic parameters are suitable to assess the organs prior to transplantation. This is most useful for a clinical setting as it allows for a rapid availability of the data.

### Blood parameters

Besides hemodynamic parameters, readily available blood parameters might also be of use as markers for organ quality. Restricted blood flow can lead to ischemia, which then leads to injury of the tissue. In a clinical setting, an adjacent lab can quickly measure routine parameters related to those injuries.

Cellular damage, assessed by blood parameters, was present at some degree in all kidneys of our cohort. This was most likely due to ischemia-reperfusion injury caused by the restoration of blood and oxygen circulation after a prolonged cut-off. The reviewed blood parameters were most likely released from damaged tubular cells [26]. Kaths et al. observed a significant difference of AST in perfusate of kidneys with a long WIT compared to those without, differing significantly starting at 5 h until the end of NMP at 7 h [8]. We found a significant difference already at 4 h. They also noted, in concordance with our results, that LDH did not differ between the groups [8]. As a cellular damage marker, the increased AST level in PT-kidneys was surprising. We expected higher levels in kidneys showing visible damage. This might be indicative of invisible damage in the PT-kidneys and will need to be elucidated.

Pool et al. tried several different erythrocyte-based perfusates during NMP and measured LDH as a marker of general injury [19]. They observed a rise of LDH levels in all perfusates during NMP. The increase was highest at the beginning, and stabilized after a few hours [19]. However, a different study also observed an increase of LDH levels during 15 h of NMP [5]. It appears that a certain amount of damage is unavoidable during ischemia-reperfusion. Furthermore, the machine itself might contribute to the increase of LDH, causing haemolysis by exerting a mechanical strain.

The anaerobic metabolite lactate was elevated after 1 h in both of our groups, as the warming up of the kidney initiated the cellular metabolism before cellular homeostasis was fully restored. Afterwards, it only decreased in PT-kidneys showing that perfusion provided sufficient oxygen to the previously anoxic tissue. Lactate levels rose continuously in NT-kidneys during NMP, indicating the lack of tissue-available oxygen. Low or declining perfusate lactate levels are one of the parameters used to identify transplantable livers during NMP [27].

Mazilescu et al. also observed decreasing lactate levels during an erythrocyte-based NMP [5]. The lactate increase was higher in non-oxygenated HMP, highlighting the importance of sufficient oxygen availability. Additionally, kidneys treated with NMP had better function at day 3 after auto-transplantation than after HMP [5]. Less injured grafts were associated with a greater lactate reduction which correlated with an improved post-operative kidney function [8].

In our setting, lactate showed the most promising results, clearly aligning with the literature as being increased in NT-kidneys. So far the other observed blood parameters were not appropriate in our setting as they showed no clear association with organ quality despite established literature.

### Limitations

While the results of our investigations look promising, limitations of this setup need to be considered. As longtime observations or auto-transplantation were not possible, conclusive evidence for the actual outcome of the kidneys with proof of functionality in a transplant recipient was not assessable. In addition to this, a single experienced physician carried out the macroscopic assessment. For this reason, a certain bias and subjectivity remains. Therefore, the implications of the parameters during whole blood NMP for long-term graft survival and function are unclear. Still, macroscopy is the leading decision factor for transplanting physicians besides donor characteristics. In a NMP setting directly measurable hemodynamic parameters and quickly available laboratory parameters can be used as an additional tool of evaluating the organ quality. In a transplantation setting, these parameters may give supporting data to aid a transplant physician in their decision-making.

## Conclusion

Besides donor characteristics, macroscopy of a kidney transplant is still the leading decision factor for transplanting physicians. It is also one of the three factors in the assessment score of machine-perfused kidneys developed by Hosgood et al. [13]. NMP can contribute to this reflecting the situation of ischemia-reperfusion before transplantation allowing to get insight of an organ during that period. We focused on markers that can easily and quickly be measured during the perfusion (hemodynamic parameters) or after perfusion in a clinic-adjacent lab (blood parameters). During NMP directly available hemodynamic parameters were strongly associated with the macroscopy and can potentially be used as quality criteria as well. The quickly determinable blood marker lactate supported the macroscopic classification. Nevertheless, to predict organ quality and damage in marginal kidneys additional molecular markers and biopsies are still necessary. For this, the NMP with whole blood is suitable platform as it allows for a good model of ischemia-reperfusion injury. Additionally, confirmation of the data in a transplant model as well as a later transfer to a clinical setting are still required.

## Electronic supplementary material

Below is the link to the electronic supplementary material.

Supplementary Material 1

## Data Availability

All data is available upon request from the authors.
