# Supplementary material for: Assessment of hemodynamic and blood parameters that may reflect macroscopic quality of porcine kidneys during normothermic machine perfusion using whole blood
Source: World J Urol. 2024 Aug 7;42(1):471. doi: 10.1007/s00345-024-05139-2 (PMC11306647; doi:10.1007/s00345-024-05139-2)
Supplement: Supplementary file 1 — Supplementary Material 1 [file 345_2024_5139_MOESM1_ESM.pptx]

## Slide 1
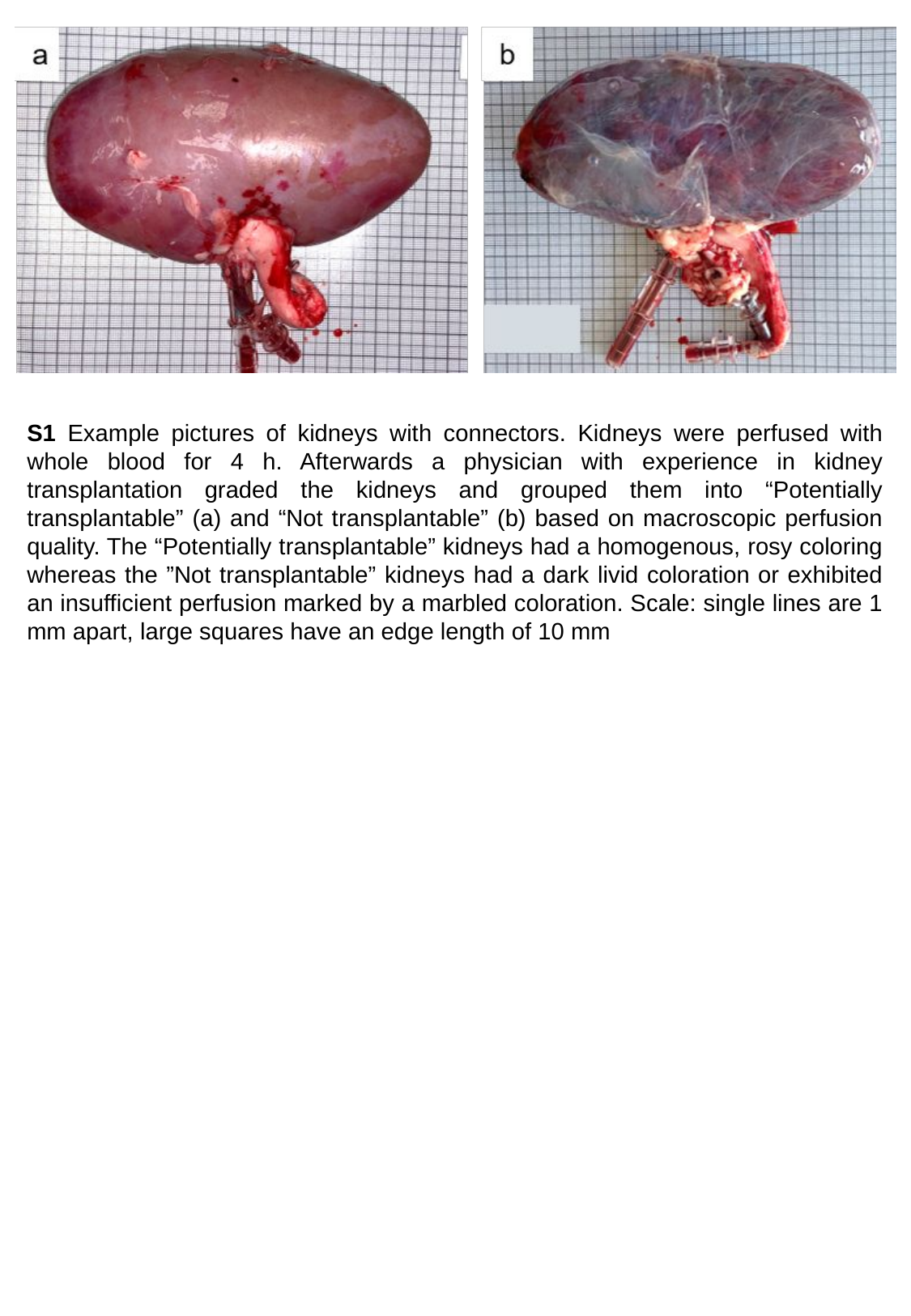

S1 Example pictures of kidneys with connectors. Kidneys were perfused with whole blood for 4 h. Afterwards a physician with experience in kidney transplantation graded the kidneys and grouped them into “Potentially transplantable” (a) and “Not transplantable” (b) based on macroscopic perfusion quality. The “Potentially transplantable” kidneys had a homogenous, rosy coloring whereas the ”Not transplantable” kidneys had a dark livid coloration or exhibited an insufficient perfusion marked by a marbled coloration. Scale: single lines are 1 mm apart, large squares have an edge length of 10 mm
